# Promising Antiparasitic Natural and Synthetic Products from Marine Invertebrates and Microorganisms

**DOI:** 10.3390/md21020084

**Published:** 2023-01-25

**Authors:** Mingyue Zhang, Qinrong Zhang, Qunde Zhang, Xinyuan Cui, Lifeng Zhu

**Affiliations:** 1 College of Life Sciences, Nanjing Normal University, Nanjing 210046, China; 2 School of Medicine & Holistic Integrative Medicine, Nanjing University of Chinese Medicine, Nanjing 210023, China

**Keywords:** bioactive compound, antiparasitic drugs, marine sponges, cnidaria, bryozoa, marine bacteria, marine fungi, cyanophyta

## Abstract

Parasitic diseases still threaten human health. At present, a number of parasites have developed drug resistance, and it is urgent to find new and effective antiparasitic drugs. As a rich source of biological compounds, marine natural products have been increasingly screened as candidates for developing new antiparasitic drugs. The literature related to the study of the antigenic animal activity of marine natural compounds from invertebrates and microorganisms was selected to summarize the research progress of marine compounds and the structure–activity relationship of these compounds in the past five years and to explore the possible sources of potential antiparasitic drugs for parasite treatment.

## 1. Introduction

Parasitic diseases common in the tropics and subtropics, including malaria, leishmaniasis, trypanosomiasis, and others, still threaten the lives and property of indigenous people [1].

Malaria, which occurs mainly in sub-Saharan Africa [2], is caused by *Plasmodium*. *Anopheles gambiae* is the principal vector of the disease in the Afrotropical Region [3]. *Plasmodium* enters human liver cells via infected female *Anopheles* and proliferates. Then, merozoites invade red blood cells and further cause disease [4], which is characterized by fever, headache, vomiting, diarrhea, chills, and muscle aches [5]. According to the World Health Organization, an estimated 240 million malaria cases were endemic in 84 countries worldwide in 2021 [6].

Leishmaniasis and trypanosomiasis are neglected tropical diseases (NTDs) that are associated with extreme poverty [7], spread in tropical and subtropical areas in 149 countries, and affect more than 2 billion poor people worldwide [8].

Leishmaniasis is affected by poor nutrition, poor sanitation, a weak immune system, and a lack of preventive measures [9]. This parasite occurs in Asia, Africa, the Americas, and the Mediterranean region. The main genera responsible for this disease are *Phlebotomus* and *Lutzomyia* [10]. Sand flies bite an infected animal host and acquires *Leishmania*, which multiplies in the gut. After 8 to 20 days, they become infectious and spread the disease by biting other hosts [11]. Leishmaniasis includes cutaneous leishmaniasis (CL), visceral leishmaniasis (VL), and mucocutaneous leishmaniasis (MCL). CL is the most common form, while VL is the most severe and is characterized by fever, weight loss, enlargement of the spleen and liver, and anemia [12]. Currently, the only effective treatment for leishmania is pentavalent antimony [10].

Trypanosomiasis includes sleeping sickness and Chagas disease (American trypanosomiasis); sleeping sickness is common in 36 sub-Saharan African countries [13] and is transmitted by blood-sucking tsetse flies. This parasite has two main forms: the slower-progressing form caused by *Trypanosoma brucei gambiense* and the faster-progressing form caused by *Trypanosoma brucei rhodesiense* [14]. A prominent feature of African trypanosomiasis is lethargy. *T. brucei* can circulate freely in the host’s blood and tissue fluids until it reaches the central nervous system, where it is usually fatal. Therefore, therapeutics at this stage must cross the blood–brain barrier [4]. American trypanosomiasis occurs in the Americas (including Mexico, Central, and South America) and is caused by *Trypanosoma cruzi*, which is transmitted through reduviid bugs [15,16].

Because of the widespread use of drugs, many parasites have developed resistance to treatment. For example, artemisinin-based combination therapy (ACT), which combines artemisinin and quinolines [17], is considered a first-line treatment for *Plasmodium falciparum* malaria globally [18]. Unfortunately, in the Greater Mekong subregion, such as Cambodia, Thailand, and Myanmar, the efficacy of artemisinin derivatives and ACT partner drugs is decreasing [19,20,21,22]. Additionally, the parasite has resistance to inexpensive drugs such as chloroquine and sulfadoxine/pyrimethamine. Similar situations were also observed with praziquantel for the treatment of schistosomiasis infection [23] and ivermectin for worms [24]. In addition to drug resistance, the efficacy and toxicity of drugs also deserve attention. Benznidazole and nifurtimox, which are used to treat *Trypanosoma cruzi* infection, are highly toxic to adult patients and have low efficacy [25]. Moreover, although a large number of resources have been invested, no effective vaccine against parasitic diseases has been developed thus far [26]. These reasons are forcing researchers to find new safe and effective antiparasite drugs.

The ocean covers more than 70% of the Earth’s surface area. Plants and animals, approximately 500,000 species in approximately 28 phyla, exist in this environment [27]. Compared with the terrestrial environment, the ocean has much richer biodiversity. The marine environment is more complex, and marine organisms have been in a harsh environment of high salinity, high pressure, lack of oxygen, limited food supply, and lack of photosynthesis for a long time [28]. Some organisms have evolved adaptations that allow them to synthesize toxic compounds or acquire toxic compounds from others. These toxic compounds can help protect marine life from predators [29]. Marine natural products are bioactive metabolites extracted from marine organisms, including marine animals, plants, and microorganisms [30]. Therefore, the ocean is an important source of bioactive compounds. Currently, compounds isolated from marine organisms mainly include terpenoids, alkaloids, polyketones, steroids, peptides, lactones, and so on [27,31], which have effective antibacterial, antifungal, anti-inflammatory, antiviral, antiparasitic, and other bioactivities [32,33].

We searched the Web of Science database from January 2017 to November 2022 for references with the keywords “marine-derived natural antiparasite products” and further screened the relevant research literature on invertebrates and microorganisms. We did not include meetings or review articles. In this review, we also used the following criteria to determine the activity of compounds:

1. When IC_50_ > 20 μM, the activity of the compounds was low or inactive; when 1 ≤ IC_50_ ≤ 20 μM, the compounds showed moderate activity. When IC_50_ < 1 μM, they showed good potent activity [34];
2. When measured in μg/mL, if IC_50_ > 20 μM, the activity of the compounds was low or inactive; if 3 ≤ IC_50_ ≤ 10 μg/mL, the compound showed moderate activity. If IC_50_ < 3 μg/mL, the compound showed good potent activity [35].

**Figure 1:**
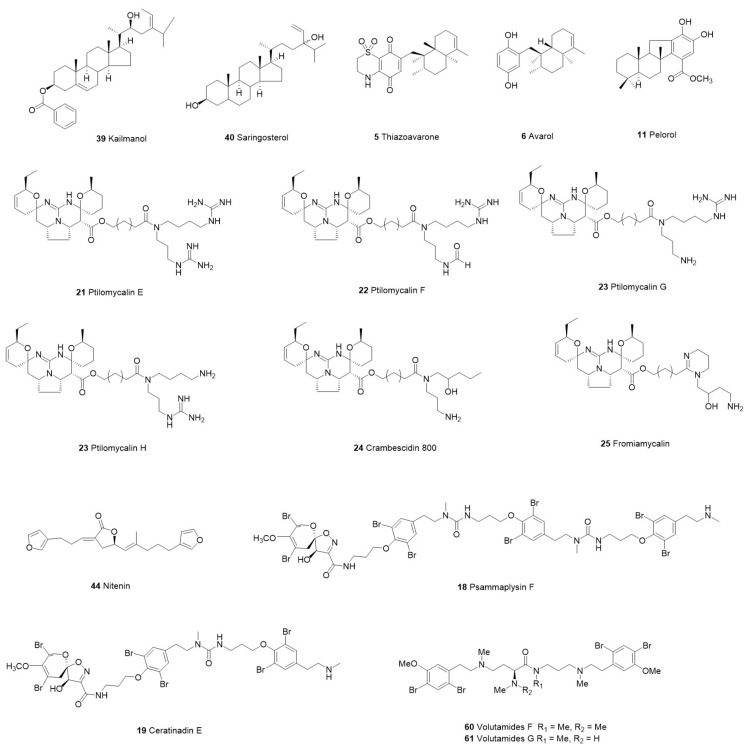
The structure of compounds with effective antiparasitic activity in invertebrates.

**Figure 2:**
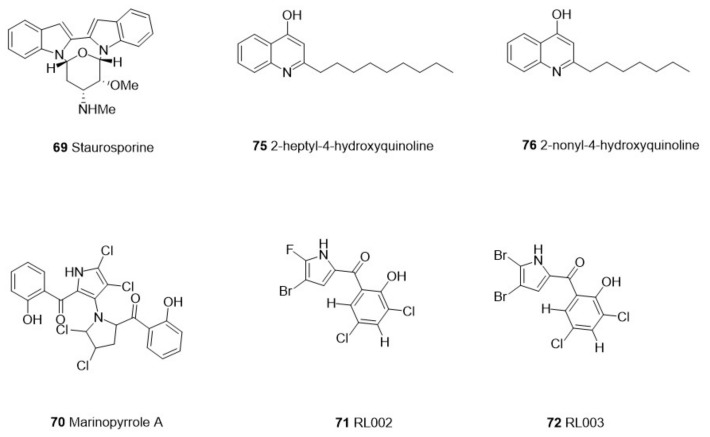
The structure of compounds with effective antiparasitic activity in marine bacteria.

**Figure 3:**
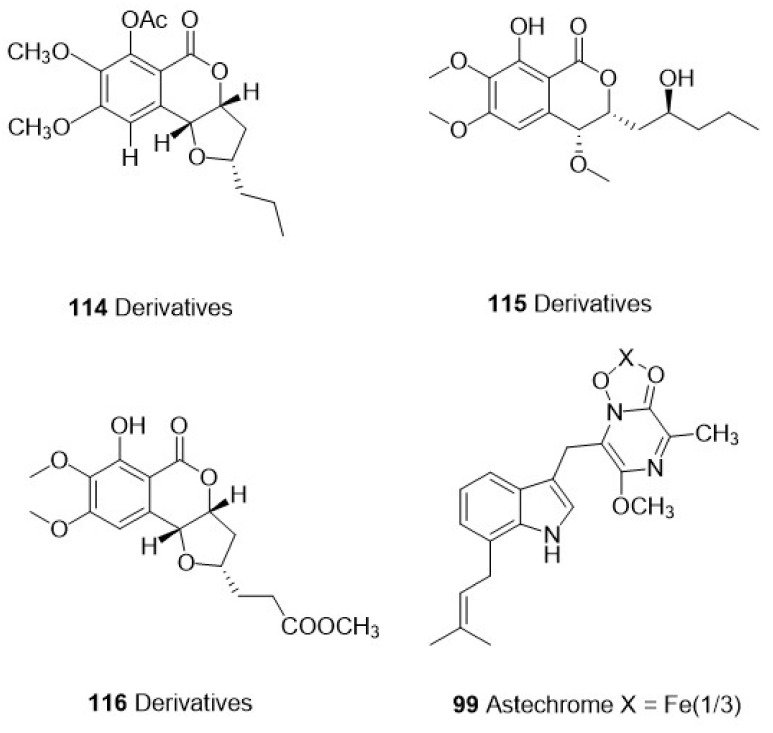
The structure of compounds with effective antiparasitic activity in marine fungi.

**Figure 4:**
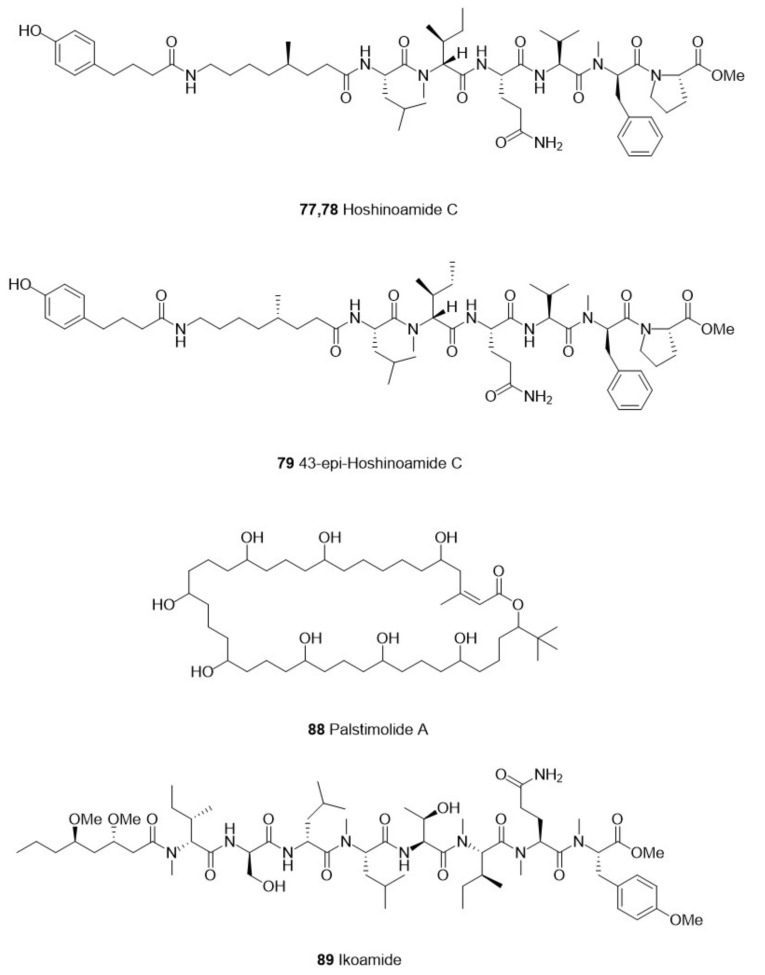
The structure of compounds with effective antiparasitic activity in Cyanophyta.

We screened 36 studies on the derivatives from invertebrates and microorganisms (Table 1) and six studies on their crude extracts (Table 2). We reviewed the literature on the purification of the derived compounds. Twelve invertebrate marine sponges came from 11 genera: *Aplysinella*, *Dysidea*, *Fascaplysinopsis*, *Hyrtios*, *Ircinia*, *Pseudoceratina*, *Monanchora*, *Mycale*, *Tedania*, and *Xestospongia*. Five genera, *Bebryce*, *Macrorhynchia*, *Plumarella*, and *Sinulari*, were included in the seven studies regarding cnidarians. Two genera, *Amathia* and *Orthoscuticella*, were involved in two bryozoan studies. For microorganisms, two genera, including *Streptomyces* and *Pseudomonas*, were studied in three bacterial studies. *Aspergillus*, *Cochliobolus*, *Exserohilum*, and *Paecilomyces* were involved in four fungal studies. Nine cyanobacteria studies involved *Caldora, Dapis, Leptolyngbya, Okeania, Salileptolyngbya,* and *Moorea*. Finally, we summarized the chemical structures with good potent activity (Figure 1, Figure 2, Figure 3 and Figure 4) and the possible structure–activity relationships.

## 2. Marine Invertebrate-Derived Antiparasitic Compounds

Invertebrates make up a large part of the literature collected on antiparasitic compounds of marine origin (58.33%). Most of these compounds are alkaloids (including bromotyrosine alkaloids, tryptophan-derived alkaloids, acyclic guanidine alkaloids, etc.), sesquiterpenoids, diterpenoids, sterols, steroids, etc. (Table 1). Invertebrate-derived compounds against *P. falciparum* have highly effective bioactivity (Figure 1).

### 2.1. Alkaloid Compounds

Bromopyrrole alkaloids are a field worth exploring for antiparasitic drugs [46]. The bromotyrosine alkaloid bisaprasin (**3**) extracted from marine sponges was moderately effective against *T. cruzi* (IC_50_ = 0.61 µM) [36]. Pseudoceratidine (1) (**29**) and its derivatives extracted from *Tedania brasiliensis* have moderate efficacy against *P. falciparum*, *L. infantum*, *L. amazonensis*, and *T. cruzi* (Table 1). The antiplasmodium activity of this alkaloid is related to the length of the polyamine chain containing basic nitrogen and the presence of bromine atoms on the terminal portion of pyrrole or furan. Moreover, Parra et al. [46] found that pseudoceratidine (1) (**29**) had additive effects when used in combination with artesunate. Consequently, pseudoceratidine (1) (**29**) can be used as a promising source of antiplasmodial drugs.

Campos et al. [44] extracted pentacyclic alkaloids (ptilomycalin E, ptilomycalin F, and ptilomycalins G+H (**21**–**23**)) and acyclic guanidine alkaloids (crambescidin 800 (**24**) and fromiamycalin (**25**)) from *Monanchora unguiculata* sponges, which have extremely high activity against the chloroquine-sensitive 3D7 strain of *P. falciparum* (IC_50_ were 0.35, 0.23, 0.46, 0.52, and 0.24 µM, respectively) [82]. The antimalarial activity of pentacyclic alkaloids is related to their five-ring structure. Unguiculin A (**20**), which has no five-ring structure, has lower antimalarial activity (IC_50_ = 12.89 µM). Ceratinadin E (**19**), a new bromotyrosine alkaloid, was isolated from the marine sponge *Pseudoceratina* by Kurimoto et al. [43] and showed good potent activity against the chloroquine-resistant strain FCR3 (IC_50_ = 0.77 µg/mL) and multidrug-resistant strain K1 (IC_50_ = 1.03 µg/mL) of *P. falciparum*. In 2019, Campos et al. [38] isolated 8-oxo-tryptamine (**7**) and the mixture of (E) with (Z)-6-bromo-2′-demethyl-3′-N-methylaplysinopsin (**8**), which showed moderate activity against the *P. falciparum* 3D7 strain ((IC_50_ were 8.8 and 8.0 µg/mL, respectively). These two aplysinopsins with antimalarial activity have a double bond between C-8 and C-1′, suggesting that antimalarial activity may be connected to the skeleton of the compounds.

Brominated alkaloids extracted from the bryozoan *Amathia lamourouxi* showed effective antimalarial activity against the *P. falciparum* 3D7 strain. Moreover, volutamide F (**60**) showed a higher selectivity index for the human embryonic kidney cell line HEK293. The antimalarial activity of volutamide H (**62**) (IC_50_ = 1.6 µM) was lower than that of volutamide F (**60**) (IC_50_ = 0.61 μM) and volutamide G (**61**) (IC_50_ = 0.57 µM), indicating that the presence of tertiary amides plays an important role against *Plasmodium* [55]. Alkaloids (orthoscuticellines A, D, E, 1-ethyl-β-carboline (**63, 65, 66, 68**)) isolated from *Orthoscuticella ventricosa*, another bryophyte, also had moderate antimalarial activity, ranging from 12–21 μM (Table 1). Ligand efficiency calculations showed that β-carboline was partly related to the antiplasmodium activity [56].

### 2.2. Terpenoids, Sesquiterpenoids, and Diterpenoids Compounds

Imperatore et al. [37] obtained the natural sesquiterpenoid quinone avarone (**4**) and avarol (**6**) from *Dysidea avara* sponges. They obtained the semisynthetic thiazinoquinone derivative thiazoavarone (**5**) by condensation reaction of avarone (**4**) with subtaurine. Compared with the two natural products, thiazoavarone (**5**) showed better activity against the chloroquine-resistant strain W2 (IC_50_ = 0.21 μM) and drug-sensitive strain D10 (IC_50_ = 0.38 μM) of *P. falciparum*. In addition, this derivative also had bioactivity against *Schistosoma mansoni* (IC_50_ = 5.90 μM). These results suggested that the substituent of the 1,1-dioxo-1,4-thiazine ring played a vital part in bioactivity.

Among the five new furan diterpenes keikipukalides (A–E) (**46**–**50**) isolated from *Plumarella delicatissima*, four keikipukalides (B–E) (**47**–**50**) showed moderate activity against *L. donovani* (IC_50_ were 8.5, 8.8, 12, and 8.8 μM, respectively). In addition, the two known compounds pukalide (**51**) and norditerpenoid (**52**) ineleganolide that were isolated, also showed good biological activity (IC_50_ were 1.9 and 4.4 μM, respectively). In particular, these compounds were not toxic to human lung carcinoma cells when they were below 50 μM. [52]. The sesquiterpenoids alcyopterosin V (**41**) and alcyopterosin E (**42**) obtained from another cnidarian *Alcyonium* sp. also had moderate activity against *L. donovani* (IC_50_ were 7.0 and 3.1 μM, respectively) [49].

### 2.3. Steroids and Sterols Compounds

Chlorinated steroid (3) (**53**), 24-methylenecholestane-3β-5α,6β-triol-6-monoacetate (**55**), and dibromoditerpene compounds pinnaterpene C (**54**) extracted from *Sinularia brassica* at 50 μM showed positive effects. The inhibitory effects of *L. donovaniamastigote* on amastigotes were 58.7%, 54.7%, and 74.3%, respectively. In addition, the three compounds showed little toxicity to THP-1 cells at these concentrations [53].

Two sterol compounds, kaimanol (**39**) and saringosterol (**40**), were extracted from the sponge *Xestospongia* sp. The antimalarial activity of kaimanol (**39**) was lower than that of saringosterol (**40**), suggesting that benzoyl may reduce the activity in the sterol structure [47]. The terpenoids extracted from the sponge *Hyrtios erectus* and the cnidarian *Bebryce grandis* showed moderate or greater activity against chloroquine-resistant Dd2 strains [39,50]. It is worth noting that both compounds extracted from *B. grandis* act on the life cycle of *Plasmodium* parasites. They found that the addition of nitenin (**44**) before the ring transition to the early trophozoite stage inhibited the maturation of the parasites. Bebrycin A (**43**) prevented the parasite from maturing. Among the clinical antimalarial drugs, only artemisinin is active against the merozoite of *Plasmodium* [83]. Consequently, Wright et al. [50] noted that it might be possible to develop new artemisinin combination therapy partner drugs based on the properties of these two terpenoids.

### 2.4. Other Compounds

Sala et al. extracted several nitrile-containing polyacetylene secondary metabolites from the sponge *Mycale* sp.SS5; however, only albanitrile A (**26**) showed moderate bioactivity against *Giardia duodenalis* (IC_50_ =12 μM). The lower bioactivity of albanitrile B (**27**) than A **26** also suggested that the activity of antigenic animals depended on the chain length of the alkyl group [45].

Notably, isololiolide (**45**), which was extracted in the sponge *Macrorhynchia philippina*, had certain effects on *T. cruzi* trypomastigotes and amastigotes (IC_50_ = 31.9 and 40.4 μM, respectively). Lima et al. [51] studied the lethal mechanism of this compound and suggested that isololiolide (**45**) may cause damage to plasma membrane integrity and depolarization of mitochondrial membrane potential.

## 3. Marine Microorganisms-Derived Antiparasitic Compounds

### 3.1. Steroids and Sterols Compounds

Previous studies have shown that polyketones, alkaloids, fatty acids, terpenes, and other compounds isolated from marine bacteria have potential antibacterial, antifungal, and antiparasitic activities [74,84,85]. *Salinivibrio* and *Streptomyces* from Actinomycetes are Gram-positive bacteria [74], while *Pseudomonas* from Proteobacteria is Gram-negative bacteria [86]. The active compounds extracted from these bacteria mainly include alkaloids and quinoline (Table 1) (Figure 2).

Marinopyrrole A (**70**), an alkaloid compound found in marine *Streptomyces* sp., has strong antibacterial activity against methicillin-resistant *Staphylococcus aureus* [87]. Martens et al. [58] explored the activity of this compound against *Toxoplasma gondii*. In in vitro experiments, marinopyrrole A (**70**) showed potent inhibitory activity at 0.31 µM against *Toxoplasma gondii* tachyzoites. However, the anti-toxoplasma effect was inhibited when more than 20% bovine calf serum was added to the liquid medium. Based on compound (**70**), they obtained three analogs, RL002, RL003, and RL125 (**71**–**73**), which showed 3.6- to 6.8-fold increased efficacy against toxoplasmosis (*P* < 0.001, Student’s paired *t*-test) and decreased serum sensitivity. RL003 (**72**), the most inhibitory analog, is highly active against cysts in vitro (IC_50_ = 0.245 μM). Hence, further in vivo chronic studies are needed to assess the potential antiparasitic activity of RL003 (**72**) in the host. Another alkaloid, staurosporine (**69**), isolated from *Streptomyces* sp. PBLC04 can kill the trophozoites of *Acanthamoeba* (IC_50_ = 0.265 µg/mL) [57]. The cysts of *Acanthamoeba* allow the parasite to cope with harsh environments such as a lack of nutrients, high temperatures, and high osmotic pressure, so *Acanthamoeba*, in this stage is highly resistant [88,89]. Notably, taurosporine also showed good potent inhibition against cysts (IC_50_ = 0.771 μg/mL). The protein kinase family is generally considered to be the main target of staurosporine (**69**) [90]. *Acanthamoeba* is rich in known kinase genes, which may explain the high activity of this compound against *Acanthamoeba*.

Martinez-Luis et al. [59] isolated five hydroxyquinoline compounds from *Pseudomonas aeruginosa*, among which three compounds had good antiparasitic effects: 3-heptyl-3-hydroxy-1,2,3,4-tetrahydroquinoline-2.4-dione (2), 2-heptyl-4-hydroxyquinoline (3), and 2-nonyl-4-hydroxyquinoline (4) (**74**–**76**). These three compounds showed moderate and greater antimalarial activity against the chloroquine-resistant strain W2 of *P. falciparum* (IC_50_ = 3.47, 2.57, and 2.79 µg/mL, respectively). Compounds (3) (**75**) (IC_50_ = 3.66 µg/mL) and (4) (**76**) (IC_50_ = 3.99 µg/mL) also showed resistance to *Trypanosoma cruzi*. In addition, this study also found that the corresponding tautomers of compounds (3) (**75**) and (4) (**76**) showed strong activity against the chloroquine-sensitive D6 strain and chloroquine-resistant *Plasmodium falciparum* W2 strain [91], indicating that the hydroxyquinoline compounds maintained antimalarial activity independently of their tautomers [59].

### 3.2. Marine Fungi

Endophytes are microfungi that reside in the internal tissues of plants without causing any immediate obvious negative effects [92,93]. Marine invertebrates, algae endophytes, or fungi found in marine sediments are also rich sources of bioactive natural products [94,95,96]. In the four studies on marine fungi from 2017 to 2022, the natural products were mostly polyketones and alkaloids (Table 1).

The compound harzialactone A (**117**) was extracted from *Paecilomyces* sp.7A22, a marine fungus isolated from sea squirts. This known polyketone compound has been isolated from *Trichoderma harzianum*, an endophytic fungus of the sponge *Halichondria okadai* [97]. Braun et al. [72] investigated the antiparasitic activity of this polyketone compound.

Harzialactone A (**117**) had the ability to overcome the transmembrane barriers to reach the macrophage phagolysosome, where amastigotes grow, and showed moderate activity against *L. amazonensis promastigotes* (IC_50_ = 5.25 μg/mL). In addition, another polyketone isolated from *Cochliobolus lunatus* by Xu et al. [70] (Derivatives **103**–**111**, Acyl derivatives **69**–**71**) showed moderate antiplasmodial activity (Table 1). The structure–activity relationships showed that biphenyl substituents at C-2, acetone at C-5′ and C-6′, and triple or quadruple substitution of acyl groups increased antiplasmodium activity.

Isocoumarins (1) (**112**) and isocoumarins (3) (**113**) extracted from *Exserohilum* sp. (CHNSCLM-0008) fungus isolated from button coral *Palythoa haddoni* by Coronado et al. [71] showed moderate activity against chloroquine-sensitive HB3 strains of *Plasmodium falciparum* (IC_50_ values were 1.13 and 11.7 μM, respectively). Semisynthetic derivatives were obtained by changing the substituents of the aromatic ring and adipose chain to explore the structure–activity relationship of the compounds. The newly synthesized compounds, derivatives **114**–**116** (Figure 3), showed good potent activity against *P. falciparum* (IC50 values were 0.77, 0.38, and 2.58 μM, respectively). Among them, derivative **115** was an accidental ring-opening product obtained during the demethylation process, which had a very strong antimalarial effect. Moreover, structure–activity analysis demonstrated that the configuration of methoxy groups and *3R*, *4R*, and *10S* was necessary for antimalarial activity, and the lipid solubility of the side chain could help improve antimalarial activity. On the one hand, derivative **115** can inhibit heme polymerization and reduce mitochondrial membrane potential in the parasite; on the other hand, they can inhibit DNA gyrase enzymes and thus inhibit DNA replication. In conclusion, this study suggested that derivatives **115** may be a potential lead agent for malaria treatment.

Bunbamrung et al. [69] isolated the fungus *Aspergillus terreus* BCC51799 from decaying wood samples in the ocean and extracted new natural products from this fungus. Among them, the alkaloid astechrome (**99**) (Figure 3) showed strong antimalarial activity (IC_50_ = 0.94 μM) (Table 1).

## 4. Cyanophyta

Cyanobacteria, also known as blue-green algae because of the presence of phycocyanin and chlorophyll, are the only prokaryotes that can produce oxygen through photosynthesis [98]. Some secondary metabolites in marine cyanobacteria have good activity and are considered lead compounds for drugs [99]. Some of these compounds are antimicrobial peptides, and cyanobacterial peptides can be divided into linear peptides, depsipeptides, and cyclic peptides according to their structure [98].

### 4.1. Linear Peptides

Ozaki et al. [66] isolated the linear peptides mabuniamide (1) (**90**) and stereoisomer **2** (**91**), from *Okeania* sp., which showed moderate activity (IC_50_ were both 1.4 μM) against the chloroquine-sensitive 3D7 strain of *P. falciparum*. In 2020, Iwasaki et al. [65] isolated another linear peptide, ikoamide (1) (**89**) (Figure 4), from *Okeania* and discovered strong activity against the *P. falciparum* 3D7 strain. Kurisawa et al. [62] isolated three linear peptides from the cyanobacteria *Dapis* sp. However, only iheyamides A (**86**) showed moderate activity against *T. b. rhodesiense* (IC_50_ = 1.5 μM) and *T. b. brucei* (IC_50_ = 1.5 μM). Structure–activity analysis proved that the C-terminal pyrrolinone moiety was vital for antiparasitic activity. The team then isolated the C-terminal part of iheyamide A (1) to obtain iheyanone (2), which also showed some activity against *T. b. rhodesiense*. To further clarify the structure–activity relationship of this compound, Iswasaki et al. [61] synthesized a variety of compounds with different peptide chain lengths and found that longer lengths of the peptide chain were more effective in inhibiting the growth of *Trypanosoma*. Hoshinoamide C (**77**) (Figure 4), a natural product discovered by Iswasaki et al. [60] in *Caldora penicillate*, also had some effective activity against *P. falciparum* (IC_50_ = 0.96 μM) and *T. b. rhodesiense* (IC_50_ = 2.9 μM). Finally, the configuration at C-43 (Figure 4) did not affect antiparasitic activity when used to synthesize two possible isomers of hoshinoamide C (**77,78**). The linear peptide Kinenzoline (1) (**92**) isolated from *Salileptolyngbya* sp. showed moderate activity (IC_50_ = 5.0 μM) against the IL-1501 strain of *T. b. rhodesiense*. Kurisawa et al. [67] also identified a synthetic pathway for kinenzoline (1) (**92**) and showed that neither natural nor synthetic Kinenzoline (1) (**92,93**) was toxic to WI-38 cells.

### 4.2. Cyclic Peptides

Cyclic peptides are likely to mimic peptide substrates or ligands of endogenous proteins (such as enzymes or receptors). Therefore, they are often considered “privileged structures” of bioactivity [100,101]. Motobamide (1) (**87**), a cyclic decapeptide isolated from *Leptolyngbya* sp., inhibited the growth of *T. b. rhodesiense*. Almaliti et al. [68] explored the relationship between the structure and activity of several dudawalamides **94**–**97**, which are cyclic depsipeptides isolated from the cyanobacterium *Moorea producens*. The results indicated that the activity of Dhoya natural products was affected by the structure of the configuration and order of residues. Keller et al. [64] isolated Palstimolide A (**88**), a polyhydroxy macrolide compound from cyanobacteria, with an IC_50_ of 0.1725 μM against the Dd2 strain of P. falciparum, showing very high antiplasmodium activity. This compound also showed moderate activity against the promastigotes phase of *L. donovani* (IC_50_ = 4.67μM).

## 5. Conclusions

Our review of the literature published in the last five years found that sponges are still the major source of marine-derived compounds. Marine sponge-derived compounds have shown excellent activity against *Plasmodium falciparum* in in vitro studies. A total of 40 natural products or synthetic compounds from marine sponges were included in this study, among which 12 compounds had good potent activity. These sponges belong to *Xestospongia*, *Dyside*, *Hyrtios*, *Pseudoceratina*, and *Monanchora*. Approximately 17 compounds were derived from cnidarians, and one compound from *Bebryce* showed good potent activity. In addition, 11 compounds from bryophytes and two high bioactivity compounds were derived from *Amathia*. A total of 8 compounds from marine bacteria were collected, and seven compounds with effective bioactivity were extracted from *Streptomyces*, *Salinivibrio,* and *Pseudomonas*. Twenty compounds were identified from marine fungi, with three highly active compounds from *Exserohilum* and *Aspergillus.* Finally, 21 were derived from Cyanophyta, with 4 highly active compounds from *Caldora*, *Okeania*, and *Leptolyngbya*.

Naturally derived or semisynthetic molecular analogs can be developed by structure–activity relationship (SAR) analysis and tend to have higher bioactivity and less toxicity [102]. In addition, it has been shown that coupling natural products with nanomaterials may enhance the activity of compounds. Walvekar et al. used silver nanoparticles coupled with extracts of *Kappaphycus alvarezii*, which enhanced anti-acanthamoebic activity [103].

Although the association between the structure of some compounds and their antiparasitic activity has been explored through SAR, the molecular targets and mechanisms of some compound molecules have not been clarified [104]. At present, a large number of promising active antiparasitic compounds have been discovered, but translating them into a drug for clinical use still faces many difficulties: (1) if the purified antiparasitic product is not chemically synthesized, clinical studies and mass production of those compounds often require more biomass than discovering new compounds and (2) if the compounds can be obtained through chemical synthesis, it is also worth considering how to reduce the synthesis steps and reduce the cost of chemical synthesis.

## Figures and Tables

**Table 1:** Natural products or derivatives from marine invertebrates and microorganisms.

Category		Species	Compounds	Chemistry	Target Parasite	Stage/Strain	IC_50_	Cytotoxicity		Site	Reference
								Type of Cells	IC_50_		
Invertebrate	sponges	*Aplysinella rhax*	**1** Psammaplin A	Bromotyrosine Alkaloids	*T. cruzi*	C2C4	30 μM	NT	NT	Fiji Islands	[36]
					*P. falciparum*	3D7	60 μM				
			**2** Psammaplin D		*T. cruzi*	C2C4	43 μM				
					*P. falciparum*	3D7	67 μM				
			**3** Bisaprasin		*T. cruzi*	C2C4	19 μM				
					*P. falciparum*	3D7	29 μM				
			Benznidazole *	-	*T. cruzi*	C2C4	2.6 μM	-	-	-	
			Chloroquine *	-	*P. falciparum*	3D7	0.017 μM				
		*Dysidea avara*	**4** Avarone	Sesquiterpene Quinone Avarone	*P. falciparum*	D10	2.74 μM	Human microvascular endothelial cells, HMEC-1	62.19 μM	Bay of Izmir, Turkey	[37]
						W2	2.09 μM				
						3D7 elo1-pfs16-CBG99	15.53 μM				
					*L. infantum*	promastigote	28.21 μM	Human acute monocytic leukemia cells, THP-1	>100 μM		
					*L. tropica*	promastigote	20.28 μM				
					*L. infantum*	amastigotes	7.64 μM				
					*S. mansoni*	schistosomula	42.77 μM				
			**5** Thiazoavarone		*P. falciparum*	D10	0.38 μM	Human microvascular endothelial cells, HMEC-1	3.31 μM		
						W2	0.21 μM				
						3D7 elo1-pfs16-CBG99	15.01 μM				
					*L. infantum*	promastigote	8.78 μM	Human acute monocytic leukemia cells, THP-1	7.41 μM		
					*L. tropica*	promastigote	9.52 μM				
					*L. infantum*	amastigotes	4.99 μM				
					*S. mansoni*	schistosomula	5.90 μM				
			**6** Avarol		*P. falciparum*	D10	0.96 μM	Human microvascular endothelial cells, HMEC-1	36.85 μM		
						W2	1.10 μM				
						3D7 elo1-pfs16-CBG99	9.30 μM				
					*L. infantum*	promastigote	7.42 μM	Human acute monocytic leukemia cells, THP-1	31.75 μM		
					*L. tropica*	promastigote	7.08 μM				
					*L. infantum*	amastigotes	3.19 μM				
					*S. mansoni*	schistosomula	33.97 μM				
			Chloroquine *	-	*P. falciparum*	D10	0.04 μM	-	-	-	
						W2	0.54 μM				
			Methylene blue *	-		3D7 elo1-pfs16-CBG99	0.155 μM				
			Amphotericin B *	-	*L. infantum*	promastigote	0.2 μM				
					*L. tropica*	promastigote	0.17 μM				
					*L. infantum*	amastigotes	0.189 μM				
		*Fascaplysinopsis reticulata*	**7** 8-oxo-tryptamine	Tryptophan-Derived Alkaloids	*P. falciparum*	3D7	8.8 µg/mL	NT	NT	Mayotte	[38]
			**8** The mixture of the known (E) and (Z)-6-bromo-2′-demethyl-3′-N-methylaplysinopsin				8.0 µg/mL				
			Artemisinin *	-			0.006 μg/mL	-	-	-	
		*Hyrtios erectus*	**9** Smenotronic acid	Sesquiterpenoids	*P. falciparum*	Dd2	3.51 μM	NT	NT	Sesquiterpenoids	[39]
			**10** Ilimaquinone				2.11 μM				
			**11** Pelorol				0.80 μM				
		*Hyrtios* sp.	**12** Hyrtiodoline A	Alkaloid	*T. brucei* *brucei*	-	48 h: 15.26 μM	J774.1 macrophages	>200 μM	Red Sea at Sharm el-Sheikh, Egypt	[40]
							72 h: 7.48 μM				
		*Ircinia oros*	**13** Ircinin-1	Linear Furanosesterterpenoids	*T. b. rhodesiense*	-	97 μM	L6 rat myoblast cells	150 μM	Gökçeada, Northern Aegean Sea, Turkey	[41]
					*T. cruzi*		120 μM				
					*L. donovani*		31 μM				
					*P. falciparum*		58 μM				
			**14** Ircinin-2		*T. b. rhodesiense*		65 μM		140 μM		
					*T. cruzi*		110 μM				
					*L. donovani*		28 μM				
					*P. falciparum*		56 μM				
			**15** Ircinialactam E		*T. b. rhodesiense*		130 μM		>200 μM		
					*P. falciparum*		95 μM				
			**16** Ircinialactam F		*T. b. rhodesiense*		130 μM		>200 μM		
					*L. donovani*		95 μM				
			Melarsoprol *	-	*T. b. rhodesiense*	-	0.015 μM	-	-	-	
			Benznidazole *		*T. cruzi*		3.07 μM				
			Miltefosine *		*L. donovani*		0.51 μM				
			Chloroquine *		*P. falciparum*		0.009 μM				
			Podophyllotoxin *		*-*	-	-	L6 rat myoblast cells	0.010 μM		
		*Ircinia wistarii*	**17** Ircinianin	Sesterterpenes	*P. falciparum*	NF54	25.4 μM	HeLa	>64 μg/mL	Wistari Reef, Great Barrier Reef, Australia	[42]
					*T. brucei rhodesiense*	STIB900	82.8 μM				
					*T. cruzi*	C2C4	190.9 μM	L6	59.5 μg/mL		
					*L. donovani*	MHOM/ET/67/L82	16.6 μM				
			Chloroquine *	-	*P. falciparum*	NF54	0.006 μM	-	-	-	
			Melarsoprol *		*T. brucei rhodesiense*	STIB900	0.020 μM				
			Benznidazole *		*T. cruzi*	C2C4	3.36 μM				
			Miltefosine *		*L. donovani*	MHOM/ET/67/L82	0.486 μM				
		*Pseudoceratina* sp.	**18** Psammaplysin F	Bromotyrosine Alkaloid	*P. falciparum*	K1	3.77 µg/mL	MRC-5	12.65 µg/mL	Okinawa, Japan	[43]
						FCR3	2.45 µg/mL				
			**19** Ceratinadin E			K1	1.03 µg/mL		15.99 µg/mL		
						FCR3	0.77 µg/mL				
			Chloroquine *	-		K1	0.34 µg/mL		>25.80 µg/mL	-	
						FCR3	0.035 µg/mL				
			Artemisinin *			K1	0.010 µg/mL		>14.12 µg/mL		
						FCR3	0.0088 µg/mL				
		*Monanchora unguiculata*	**20** Unguiculin A	Acyclic Guanidine Alkaloid	*P. falciparum*	3D7	12.89 μM	KB Cells	7.66 μM	Mitsio Islands, Madagascar	[44]
			**21** Ptilomycalin E	Pentacyclic Alkaloids			0.35 μM		0.85 μM		
			**22** Ptilomycalin F				0.23 μM		1.61 μM		
			**23** Ptilomycalins G+H				0.46 μM		0.92 μM		
			**24** Crambescidin 800	Acyclic Guanidine Alkaloid			0.52 μM		1.31 μM		
			**25** Fromiamycalin				0.24 μM		1.17 μM		
			Artemisinin *	-			0.004 μM	--	-	-	
		*Mycale* sp. *SS5*	**26** Albanitrile A	Nitrile-Bearing Polyacetylenes	*Giardia duodenalis*	713	12 μM	Mammalian myeloma cell line NS-1	50 μM	Near Albany	[45]
								Normal nontumor NFF cells	100 μM		
			**27** Albanitrile B				25 μM	Mammalian myeloma cell line NS-1	50 μM		
								Normal nontumor NFF cells	100 μM		
			**28** Albanitrile C				90 μM	Mammalian myeloma cell line NS-1	180 μM		
								Normal nontumor NFF cells	90 μM		
			Metronidazole *				2.9 μM	-	-	-	
			Sparsomycin *	-	*-*	-	-	Mammalian myeloma cell line NS-1	0.55 μM		
								Normal nontumor NFF cells	1.7 μM		
		*Tedania brasiliensis*	**29** Pseudoceratidine	Bromopyrrole Alkaloids	*P. falciparum*	3D7	EC_50_ = 1 μM	Bone marrow-derived macrophages	NT	Cabo Frio, Rio de Janeiro state, Brazil	[46]
							1.1 μM				
						K1	1.1 μM				
			**30** Pseudoceratidine derivative		*P. falciparum*	3D7	EC_50_ = 6 μM				
			**31** Pseudoceratidine derivative				EC_50_ = 4 μM				
			**32** Pseudoceratidine derivative		*L. infantum*	promastigotes	EC_50_ = 24 μM		52 μM		
					*L. amazonensis*	promastigotes	EC_50_ = 19 μM				
					*T. cruzi*	epimastigotes	EC_50_ = 7 μM				
			**33** Pseudoceratidine derivative		*L. infantum*	promastigotes	EC_50_ = 19 μM		>100 μM		
					*L. amazonensis*	promastigotes	EC_50_ = 7 μM				
					*P. falciparum*	3D7	EC_50_ = 19 μM				
			**34** Pseudoceratidine derivative		*P. falciparum*	3D7	EC_50_ = 44 μM		NT		
			**35** Pseudoceratidine derivative		*L. infantum*	promastigotes	EC_50_ = 2 μM		66 μM		
					*L. amazonensis*	promastigotes	EC_50_ = 3 μM				
					*T. cruzi*	epimastigotes	EC_50_ = 24 μM				
			**36** Pseudoceratidine derivative		*P. falciparum*	3D7	EC_50_ = 7 μM		NT		
			**37** Pseudoceratidine derivative		*L. infantum*	promastigotes	EC_50_ = 20 μM		>100 μM		
					*L. amazonensis*	promastigotes	EC_50_ = 76 μM				
			**38** Pseudoceratidine derivative		*L. infantum*	promastigotes	EC_50_ = 23 μM		82 μM		
					*L. amazonensis*	promastigotes	EC_50_ = 18 μM				
					*P. falciparum*	3D7	EC_50_ = 3 μM		NT		
			Chloroquine *	-	*P. falciparum*	3D7	0.013 μM	-	-	-	
						K1	0.167 μM				
			Pyrimethamine *	-	*P. falciparum*	3D7	0.03 μM	-	-	-	
						K1	3.9 μM				
			Cycloguanil *	-	*P. falciparum*	3D7	0.010 μM	-	-	-	
						K1	0.54 μM				
			Artesunate *	-	*P. falciparum*	3D7	0.004 μM	-	-	-	
						K1	0.003 μM				
		*Xestospongia* sp.	**39** Kaimanol	Sterol	*P. falciparum*	3D7	0.359 μM	NT	NT	Indonesia	[47]
			**40** Saringosterol				0.00025 μM				
			Artemisinin *	-			5.207 × 10^−3^ nM	-	-	-	[48]
	Cnidaria	*Alcyonium* sp.	**41** Alcyopterosin V	Illudalane Sesquiterpenes	*L. donovani*	-	7.0 μM	J774.A1 macrophages	110 μM	Scotia Arc of Antarctica	[49]
								Host cell lines HEK293T	220 μM		
								Host cell lines HepG2	288 μM		
			**42** Alcyopterosin E				3.1 μM	J774.A1 macrophages	62 μM		
								Host cell lines HEK293T	570 μM		
								Host cell lines HepG2	331 μM		
			Miltefosine *	-			6.2 μM	-	-	-	
		*Bebryce grandis*	**43** Bebrycin A	Diterpene	*P. falciparum*	Dd2	EC_50_ = 1.08 μM	HepG2 human hepatocyte carcinoma cell line	EC_50_ =21.8 μM	Southeast coast of Curacao, East of Fuikbaai	[50]
			**44** Nitenin	C21 Degraded Terpene			EC_50_ = 0.29 μM		EC_50_ =18.3 μM		
		*Macrorhynchia philippina*	**45** Isololiolide	Carotenoid Isololiolide	*T. cruzi*	trypomastigotes	31.9 μM	BMM cells	>200 μM	São Sebastião Channel, Brazil	[51]
						amastigotes	40.4 μM				
			Benznidazole *	-		trypomastigotes	16.2 μM		>200 μM	-	
						amastigotes	5.3 μM				
		*Plumarella delicatissima*	**46** Keikipukalide A	Furanocembranoid Diterpenes	*L. donovani*	amastigotes	> 28 μM	Human lung carcinoma, cells, A549 cytotoxicity	>50 μM	Stanley, Falkland Islands (Islas Malvinas), in the Southern Ocean	[52]
			**47** Keikipukalide B				8.5 μM		>50 μM		
			**48** Keikipukalide C				8.8 μM		>50 μM		
			**49** Keikipukalide D				12 μM		>50 μM		
			**50** Keikipukalide E				8.8 μM		>50 μM		
			**51** Pukalide aldehyde				1.9 μM		>50 μM		
			**52** Norditerpenoid ineleganolide				4.4 μM		>50 μM		
			Miltefosine *	-			6.2 μM	-	-	-	
		*Sinularia brassica*	**53** Chlorinated steroid	Steroid	*L. donovaniamastigote*	amastigote	Inhibition of a growth of L. donovani at 50 μM = 58.7%	THP-1 cells at 50 μM	88.8%	Van Phong bay, Khanh Hoa province, Vietnam and Institute of Oceanography, Nha Trang, Vietnam	[53]
			**54** Pinnaterpene C	Dibromoditerpene			Inhibition of a growth of L. donovani at 50 μM = 74.3%		106.2%		
			**55** 24-methylenecholestane-3β-5α,6β-triol-6-monoacetate	Steroid			Inhibition of a growth of L. donovani at 50μM = 54.7%		96.1%		
			**56** Cholestane-3β-5α,6β-triol-6-monoacetate				Inhibition of growth of L. donovani at 50μM = 39.0%		92.7%		
		*Sinularia* sp.	**57** Sinuketal	Sesquiterpenoids	*P. falciparum*	3D7	80 μM	Jurkat	24.9 μM	Yongxing Island (16°50′ N, 112°20′ E) of Xisha Islands in the South China Sea	[54]
								MDA-MB-231	32.3 μM		
								U2OS	41.7 μM		
			Dihydroartemisinine *	-			10 nM	-	-	-	
	Bryozoa	*Amathia lamourouxi*	**58** Convolutamines K	Brominated Alkaloids	*P. falciparum*	3D7	1.7 μM	Human embryonic kidney cell line, HEK293	17.01 μM	Rock pools of Woolgoolga, New South Wales, Australia	[55]
			**59** Convolutamines L			3D7	11 μM		IA at 40 μM		
			**60** Volutamides F			3D7	0.61 μM		IA at 40 μM		
						Dd2	0.75 μM				
			**61** Volutamides G			3D7	0.57 μM		11 μM		
						Dd2	0.85 μM				
			**62** Volutamides H			3D7	1.6 μM		IA at 40 μM		
						Dd2	1.9 μM				
			Chloroquine *	-		3D7	0.025 μM		67% at 4 μM	-	
						Dd2	0.18 μM				
			Dihydroartemisinin *	-		3D7	0.0020 μM		IA at 0.1 μM	-	
						Dd2	0.0020 μM				
			Puromycin *	-		3D7	0.11 μM		0.81 μM	-	
						Dd2	0.068 μM				
		*Orthoscuticella ventricosa*	**63** Orthoscuticellines A	Alkaloids	*P. falciparum*	3D7	10 μM	Human embryonic kidney cell line, HEK293	10 μM	Northern NSW, Australia	[56]
			**64** Orthoscuticellines B				> 40 μM		>40 μM		
			**65** Orthoscuticellines D				14 μM		>40 μM		
			**66** Orthoscuticellines E				12 μM		>40 μM		
			**67** 1-ethyl-4-methylsulfone-β-carboline				21 μM		>40 μM		
			**68** 1-ethyl-β-carboline				18 μM		>40 μM		
			Chloroquine *	-			0.007 μM		>40 μM		
			Artesunate *	-			0.0003 μM	-	-	-	
Microorganisms	Actinomy-cetes	*Streptomyces* sp. PBLC04	**69** Staurosporine	Alkaloid	*Acanthamoeba castellanii*	Trophozoites	0.265 µg/mL	Murine macrophage J774.A1 cell line	4.076 μM	Jambelí mangrove, Ecuador	[57]
						Cysts	0.771 µg/mL				
		*Streptomyces* sp.	**70** Marinopyrrole A	Alkaloids	*T. gondii*	Tachyzoites/Type I RH	0.31 μM	Human foreskin fibroblast (HFF)	>50 μM	Marinopyrrole A was obtained from Sigma-Aldrich	[58]
								Human hepatocarcinoma (HepG2)	5.3 μM		
			**71** RL002				0.17 μM	Human foreskin fibroblast (HFF)	>50 μM		
								Human hepatocarcinoma (HepG2)	29.0 μM		
			**72** RL003				0.09 μM	Human foreskin fibroblast (HFF)	>50 μM		
								Human hepatocarcinoma (HepG2)	49.7 μM		
			**73** RL125				0.16 μM	Human foreskin fibroblast (HFF)	>50 μM		
								Human hepatocarcinoma (HepG2)	46.5 μM		
			Pyrimethamine *	-			0.61 μM	-	-	-	
	Proteobacteria	*Pseudomonas aeruginosa*	**74** 3-heptyl-3-hydroxy-1,2,3,4-tetrahydroquinoline-2.4-dione	Hydroxyquinoline	*P. falciparum*	Indochina W2	3.47 µg/mL	NT	NT	Pacific of Panama	[59]
			**75** 2-heptyl-4-hydroxyquinoline		*P. falciparum*	Indochina W2	2.57 µg/mL				
					*T. cruzi*	C4	3.66 µg/mL				
			**76** 2-nonyl-4-hydroxyquinoline		*P. falciparum*	Indochina W2	2.79 µg/mL				
					*T. cruzi*	C4	3.99 µg/mL				
			Chloroquine *	-	*P. falciparum*	Indochina W2	0.03 µg/mL	-	-	-	
			Nifurtimox *	-	*T. cruzi*	C4	1.6 µg/mL	-	-	-	
	Cyanophyta	*Caldora penicillata*	**77** Hoshinoamide C (natural)	Lipopeptide	*P. falciparum*	3D7	0.96 μM	Human cancer cells, HeLa and HL60	No cytotoxicity at 10 μM	Ikei Island, Okinawa, Japan	[60]
					*T. brucei rhodesiense*	IL-1501	2.9 μM				
			**78** Hoshinoamide C(synthetic)		*P. falciparum*	3D7	3.2 μM				
					*T. brucei rhodesiense*	IL-1501	3.7 μM				
			**79** 43-epi-hoshinoamide C(synthetic)		*P. falciparum*	3D7	0.87 μM				
					*T. brucei rhodesiense*	IL-1501	4.4 μM				
			Atovaquone *	-	*P. falciparum*	3D7	0.00096 μM	-	-	-	
			Pentamidine *	-	*T. brucei rhodesiense*	IL-1501	0.001 μM	-	-	-	
		*Dapis* sp.	**80** Iheyanone	Linear Peptides	*T. brucei rhodesiense*	IL-1501	35 μM	WI-38 cells	>50 μM	Noho Island, Okinawa, Japan	[61]
			**81** Peptides				33 μM		>50 μM		
			**82** Peptides				24 μM		>50 μM		
			**83** Peptides				15 μM		>50 μM		
			**84** Peptides				17 μM		>50 μM		
			**85** Peptides				6.2 μM		>50 μM		
			Pentamidine *	-	*T. brucei rhodesiense*	IL-1501	0.05 μM	-	--	-	
		*Dapis* sp.	**86** Iheyamides A	Linear Peptides	*T. b. rhodesiense*	IL-1501	1.5 μM	Normal human fibroblasts, WI-38 cells	18 μM	Noho Island, Okinawa, Japan	[62]
					*T. b. brucei*	221	1.5 μM				
					*T. b. rhodesiense*	IL-1501	> 20 μM		>20 μM		
					*T. b. brucei*	221	> 20 μM				
					*T. b. rhodesiense*	IL-1501	> 20 μM		>20 μM		
					*T. b. brucei*	221	> 20 μM				
			Pentamide *	-	*T. b. rhodesiense*	IL-1501	0.005 μM	-	-	-	
					*T. b. brucei*	221	0.001 μM				
		*Leptolyngbya* sp.	**87** Motobamide	Cyclic Peptide	*T. b. rhodesiense*	IL-1501	2.3 μM	WI-38 cells	55 μM	Bise, Okinawa Island, Okinawa Prefecture, Japan	[63]
								HeLa or HL60 cells	IA at 10 μM		
		*Leptolyngbya* sp.	**88** Palstimolide A	Polyhydroxy Macrolide	*P. falciparum*	Dd2	0.1725 μM	HepG2 human liver cell line	5.04 μM	Palmyra Atoll	[64]
					*L. donovani*	promastigotes	4.67 μM	B10R murine macrophages (L. donovani host cell toxicity)	>10 μM		
		*Okeania* sp.	**89** Ikoamide	Lipopeptide	*P. falciparum*	3D7	0.14 μM	HeLa cells or HL60 cells	No cytotoxicity at 10 μM	Iko-pier, Kuroshima Island, Okinawa, Japan	[65]
			Chloroquine *	-			6.9 nM	-	-	-	
			doxorubicin	-	*-*	-	-	HeLa cells	0.24 μM	-	
								HL60 cells	46 nM	-	
		*Okeania* sp.	**90** Mabuniamide	Lipopeptide	*P. falciparum*	3D7	1.4 μM	L6 myotubes	No cytotoxicity at 10–40 μM	The coast of Odo, Okinawa, Japan	[66]
			**91** Stereoisomer 2				1.4 μM				
			Chloroquine *	-			7.6 nM	-	-	-	
		*Salileptolyngbya* sp.	**92** Kinenzoline (natural)	Linear Depsipeptide	*T. b. rhodesiense*	IL-1501	5.0 μM	WI-38 cells	>20 μM	Kinenhama beach, Kagoshima, Japan	[67]
			**93** Kinenzoline (synthetic)				4.5 μM		>100 μM		
			Pentamide *	-			0.001 μM	-	-	-	
			Adriamycin *	-	*-*	-	-	WI-38 cells	0.73 μM	-	
		*Moorea producens*	**94** Dudawalamide A	Cyclic Depsipeptides	*P. falciparum*	W2	3.6 μM	H-460 human lung cancer cell line	Little to no cytotoxicity	Papua New Guinea	[68]
					*T. cruzi*	Transgenic β-galactosidase-expressing strain	12% GI (Percentage growth inhibition) at 10 μg/mL				
					*L. donovani*	WR2810	> 10 μM				
			**95** Dudawalamide B		*P. falciparum*	W2	8.0 μM				
					*T. cruzi*	Transgenic β-galactosidase-expressing strain	7% GI at 10 μg/mL				
					*L. donovani*	WR2810	> 10 μM				
			**96** Dudawalamide C		*P. falciparum*	W2	10 μM				
			**97** Dudawalamide D		*P. falciparum*	W2	3.5 μM				
					*T. cruzi*	Transgenic β-galactosidase-expressing strain	60% GI at 10 μg/mL				
					*L. donovani*	WR2810	2.6 μM				
	Ascomycetes	*Aspergillus terreus* BCC51799	**98** Astepyrazinoxide	Alkaloid	*P. falciparum*	K-1	24.82 μM	MCF-7	34.70 μM	The marine fungus was isolated from a decayed wood sample at Hat Bang Pu, Khao Sam Roi Yot National Park, Prachuap Khiri Khan Province	[69]
								NCI–H187	5.98 μM		
								Vero	15.61 μM		
			**99** Astechrome				0.94 μM	MCF-7	IA		
								NCI–H187	IA		
								Vero	7.9 μM		
			Dihydroartemisinin *	-			2.12 × 10^−3^ μM	-	-	-	
			Mefloquine *	-			0.422 μM	-	-	-	
			Ellipticine *	-	*-*	-	-	NCI–H187	9.87 μM	-	
								Vero	5.32 μM		
			Doxorubicin *	-	*-*	-	-	MCF-7	10.97 μM	-	
								NCI–H187	0.16 μM		
			Tamoxifen *	-	*-*	-	-	MCF-7	32.95 μM	-	
		*Cochliobolus lunatus* TA26-46	**100** Derivatives	14-Membered Resorcylic Acid Lactone Derivatives	*P. falciparum*	HB3	12.59 μmol/L	HUVEC	NT	Marine-derived	[70]
			**101** Derivatives				12.39 μmol/L		NT		
			**102** Derivatives				11.55 μmol/L		NT		
			**103** Derivatives				8.06 μmol/L		>100 μmol/L		
			**104** Derivatives				6.69 μmol/L		>100 μmol/L		
			**105** Derivatives				7.82 μmol/L		>100 μmol/L		
			**106** Derivatives				9.72 μmol/L		>100 μmol/L		
			**107** Derivatives				7.82 μmol/L		>100 μmol/L		
			**108** Derivatives				7.25 μmol/L		>100 μmol/L		
			**109** Acyl derivatives				9.18 μmol/L		NT		
			**110** Acyl derivatives				6.91 μmol/L		>100 μmol/L		
			**111** Acyl derivatives				3.54 μmol/L		>100 μmol/L		
			Chloroquine *	-			32.9 nmol/L	-	-	-	
		*Exserohilum* sp.	**112** Isocoumarins	Polyketide	*P. falciparum*	HB3	1.13 μM	Vero cells	87.5 μM	Zoanthid *Palythoa haddoni*	[71]
			**113** Isocoumarins				11.7 μM		124.2 μM		
			**114** Derivatives				0.77 μM		258.0 μM		
			**115** Derivatives				0.38 μM		106.3 μM		
			**116** Derivatives				2.58 μM		262.5 μM		
		*Paecilomyces* sp. 7A22	**117** Harzialactone A	Polyketone	*L*. *amazonensis*	promastigotes	5.25 μg/mL	Peritoneal macrophages	35.21 μg/mL	Ascidian Aplidiopsis sp. collected from São Sebastião Channel in Brazil	[72]
						amastigotes	18.18 μg/mL				
			Amphotericin B *	-	*L*. *amazonensis*	promastigotes	0.119 μg/mL		22.41 μg/mL	-	
						amastigotes	0.095 μg/mL				

* Positive control; NT indicates not text; IA indicates inactive.

**Table 2:** Crude extracts of marine invertebrates and microorganisms.

Category	Species	Extract Type	Target Parasite	Stage/Strain	IC_50_	Site	References
Cnidaria	*Linuche unguiculata*	Distilled water	*Giardia duodenalis*	Trophozoites, IMSS 0989:1 strain	63 µg/mL	Puerto Morelos Reef Lagoon, Mexico	[73]
Actinomycetes	*Nocardia* sp. UA 23	ISP2 medium	*Trypanosoma brucei*	TC 221	MIC, 72 h = 7.2 µg/mL	Coscinoderma mathewsi was collected from Ahia Reefs	[74]
	*Micromonospora* sp. W305	Resin, MeOH	*Antiplasmodial Activities*	Dd2	0.42 µg/mL	The microbial population associated with deep-water invertebrates	[75]
	*Nocardiopsis* sp. V671	ASE, MeOH	*Antiplasmodial Activities*	Dd2	0.88 µg/mL	The microbial population associated with deep-water invertebrates	[75]
	*Streptomyces tendae* V324	Resin, MeOH/CH_2_Cl_2_	*Antiplasmodial Activities*	Dd2	0.35 µg/mL	The microbial population associated with deep-water invertebrates	[75]
	*Streptomyces* sp. INV ACT2	Ethyl acetate	*T. gondii*	GFP-RH tachyzoites	Inhibition ≥ 80% at 120 μg/mL	Caño Aguas Negras	[76]
	*Streptomyces* sp. RM66	On ISP2, solid media with GlcNAc	*Trypanosoma brucei*	TC 221	MIC, 72 h = 4.7 µg/mL	Hurghada (Egypt)	[77]
	*Streptomyces* sp. *V881*	Resin, CH_2_Cl_2_	*Antiplasmodial Activities*	Dd2	0.062 µg/mL	The microbial population associated with deep-water invertebrates	[75]
	*Streptomyces* sp. E677	Resin, MeOH/CH_2_Cl_2_	*Antiplasmodial Activities*	Dd2	0.037 µg/mL	The microbial population associated with deep-water invertebrates	[75]
	*Unidentified actinomycete* V663	ASE, heptane	*Antiplasmodial Activities*	Dd2	0.89 µg/mL	The microbial population associated with deep-water invertebrates	[75]
Bacteroides	*Alcanivorax* sp. V174 (G-)	Resin, MeOH/CH_2_Cl_2_	*Antiplasmodial Activities*	Dd2	0.969 µg/mL	The microbial population associated with deep-water invertebrates	[75]
	*Alcanivorax* sp. V193 (G-)	Resin, MeOH/CH_2_Cl_2_	*Antiplasmodial Activities*	Dd2	1.079 µg/mL	The microbial population associated with deep-water invertebrates	[75]
	*Endozoicomonas numazuensis* H402 (G-)	Resin, MeOH/CH_2_Cl_2_	*Antiplasmodial Activities*	Dd2	0.978 µg/mL	The microbial population associated with deep-water invertebrates	[75]
	*Marinobacter* sp. V184 (G-)	Resin, MeOH/CH_2_Cl_2_	*Antiplasmodial Activities*	Dd2	1.008 µg/mL	The microbial population associated with deep-water invertebrates	[75]
	*Marinobacter* sp. V201 (G-)	Resin, MeOH/CH_2_Cl_2_	*Antiplasmodial Activities*	Dd2	1.091 µg/mL	The microbial population associated with deep-water invertebrates	[75]
	*Marinobacter* sp. V208 (G-)	Resin, MeOH/CH_2_Cl_2_	*Antiplasmodial Activities*	Dd2	1.091 µg/mL	The microbial population associated with deep-water invertebrates	[75]
Firmicutes	*Bacillus* sp. INV FIR35	Ethyl acetate	*T. gondii*	GFP-RH tachyzoites	Inhibition ≥ 80% at 48 μg/mL	Punta Betín	[76]
	*Bacillus* sp. INV FIR48	Ethyl acetate	*T. gondii*	GFP-RH tachyzoites	Inhibition ≥ 80% at 120 μg/mL	Caño Grande	[76]
	*Fictibacillus* sp. INV FIR149	Ethyl acetate	*T. gondii*	GFP-RH tachyzoites	Inhibition ≥ 80% at 1080 μg/mL	Caño Grande	[76]
	*Paenibacillus* sp. #91_7 (IN-CRY)	Waters™ Oasis^®^ HLB extraction plates, with the sorbent Oasis^®^ HLB, was equilibrated using methanol and HPLC grade water	*T. cruzi*	Tulahuen C4	97%	Isolated from marine sponges of the Erylus genus, collected in Portuguese waters	[78]
	*Penicillium citrinum* V170	Resin, MeOH/CH_2_Cl_2_	*Antiplasmodial Activities*	Dd2	1.069 µg/mL	The microbial population associated with deep-water invertebrates	[75]
	*Penicillium* sp. N161	Resin, MeOH/CH_2_Cl_2_	*Antiplasmodial Activities*	Dd2	0.266 µg/mL	The microbial population associated with deep-water invertebrates	[75]
	*Penicillium* sp. Z691	Resin, CH_2_Cl_2_	*Antiplasmodial Activities*	Dd2	0.049 µg/mL	The microbial population associated with deep-water invertebrates	[75]
	*Talaromyces rotundus* S920	Resin, MeOH/CH_2_Cl_2_	*Antiplasmodial Activities*	Dd2	0.677 µg/mL	The microbial population associated with deep-water invertebrates	[75]
	*Tritirachium* sp. V199	Resin, MeOH/CH_2_Cl_2_	*Antiplasmodial Activities*	Dd2	0.339 µg/mL	The microbial population associated with deep-water invertebrates	[75]
ɣ-Proteobacteria	*Enterococcus faecalis* #118_3 (IN-CRY)	EPA vials: Sepabeads^®^ SP207ss resin, HPLC-grade water and acetone; medium IN-CRY	*T. cruzi*	Tulahuen C4	Percentage of growth inhibition = 81%	Isolated from marine sponges of the Erylus genus, collected in Portuguese waters	[78]
	*Enterococcus faecalis* #118_3 (IN-CRY)	Duetz extraction: Waters™ Oasis^®^ HLB extraction plates, with the sorbent Oasis^®^ HLB, was equilibrated using methanol and HPLC grade water; medium IN-CRY	*T. cruzi*	Tulahuen C4	Percentage of growth inhibition = 102%	Isolated from marine sponges of the Erylus genus, collected in Portuguese waters	[78]
	*Enterococcus faecalis* #118_4 (IN-CRY)	Duetz extraction: Waters™ Oasis^®^ HLB extraction plates, with the sorbent Oasis^®^ HLB, was equilibrated using methanol and HPLC grade water; medium IN-CRY	*T. cruzi*	Tulahuen C4	Percentage of growth inhibition = 103%	Isolated from marine sponges of the Erylus genus, collected in Portuguese waters	[78]
	*Pseudoalteromonas* sp. INV PRT33	Ethyl acetate	*T. gondii*	GFP-RH tachyzoites	Inhibition ≥ 80% at 48 μg/mL	Caño Grande	[76]
Phaeophyta	*Cladostephus hirsutus*	Ethyl acetate	*T. brucei brucei*	-	27.2 μg/mL	North-west coast of Algeria	[79]
	*Cystoseira sedoides*	Hexane	*Acanthamoeba castellanii*	Trophozoite/Neff	1009 μg/mL	Tunisian coasts, Tabarka	[80]
		Ethyl acetate			860 μg/mL		
		Methanol			836 μg/mL		
	*Dictyota ciliolata*	Hexane	*Schistosoma mansoni*		Death Ratio = 100%	Espírito Santo State, Southeastern Brazil	[81]
		Chloroform			Death Ratio = 100%		
		Supercritical fluid			Death Ratio = 100%		

## Data Availability

Not applicable.
